# Primary Synovial Sarcoma of the Scrotum

**DOI:** 10.1155/2023/7839846

**Published:** 2023-12-29

**Authors:** Nourah Al-Oudah, Sara Alanazi, Sarah Saad Alotaibi, Nayef Alzahrani

**Affiliations:** ^1^Department of Pathology and Laboratory Medicine, King Abdul-Aziz Medical City, Ministry of National Guard Health Affairs, Riyadh, Saudi Arabia; ^2^Department of Surgery, King Abdul-Aziz Medical City, Ministry of National Guard Health Affairs, Riyadh, Saudi Arabia

## Abstract

The report outlines a case of synovial sarcoma in the scrotal region. A 36-year-old male presented with a scrotal swelling. The lesion was completely resected, whereas the histopathologic examination revealed a spindle cell tumor. The tumor stained positive for pancytokeratin, AE1/AE3, epithelial membrane antigen (EMA), TLE-1, CD99, and BCL-2. The cytogenetic testing showed a chromosomal translocation in the SS18 gene at 18q11.2, consistent with the diagnosis of primary synovial sarcoma. A year later, the patient developed liver, vertebrae, and lung metastasis, which was treated with systemic chemotherapy. Treatment failed to improve the hepatic lesion that was then resected, while the spine and lung lesions were followed by radiotherapy. The patient is now alive and subject to an outstanding follow-up.

## 1. Introduction

Synovial sarcoma is a malignant spindle cell soft tissue tumor of uncertain histogenesis with a variable epithelial differentiation. The most common sites (70%) are the extremities and para-articular regions [1]. However, unusual sites of involvement include the external and internal reproductive organs of both males and females, kidney, adrenal gland, retroperitoneum, stomach, small bowel, lung, heart, mediastinum, bone, CNS, and peripheral nerves [2–4]. They represent 10-15% of all sarcomas [5]. Synovial sarcoma is equally prevalent in both sexes, regardless of age [1]. The majority of the patients are adolescents or young adults [1]. Although synovial sarcoma was originally known as a synovial sarcoma due to its histologic similarity to normal synovial tissue, it has now been recognized as a misnomer based on its immunohistochemical and ultrastructural studies [6]. It is rare for synovial sarcoma to occur within the joint cavity, so those well-documented cases of intra-articular synovial sarcoma are extremely rare [7]. Synovial sarcoma is associated with translocation causing a fusion between chromosomes SSX1, SSX2, and SSX4 and chromosome SS18, occurring in more than 90% of synovial sarcoma cases leading to the formation of SSX-SS18 oncogenes [8]. The current report will discuss a case of synovial sarcoma developed in the scrotum associated with distant metastasis in a 36-year-old male.

## 2. Case Report

**Figure 1:**
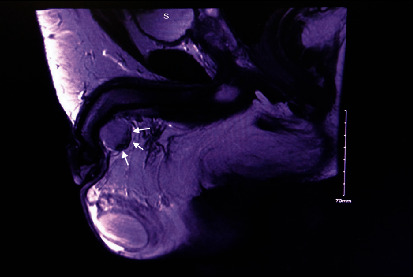
Magnetic resonance imaging finding of a mass in the left inferolateral side of the base of the penis, measuring 3.1 cm in maximum dimension.

**Figure 2:**
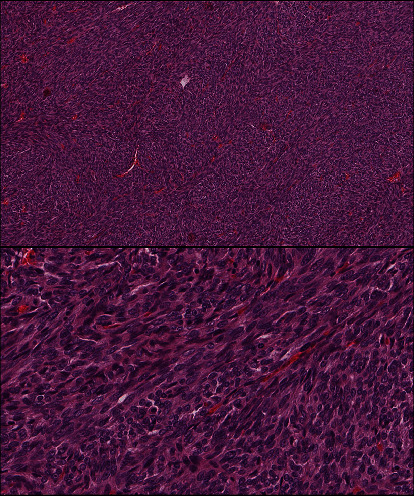
Low- and high-power images show monophasic lesion composed of spindle cells arranged in intersecting fascicles and devoid of necrosis.

A 36-year-old male presented with a scrotal swelling that lasted for five years. The scrotal mass was stable in size, but painful and interfered with his daily activities. There were no lower urinary tract symptoms nor a history of fever or weight loss. He had no history of masses or sexually transmitted diseases. Upon examination, the swelling was tender and measured 2 cm in diameter. It was attached to the penis on the left side at the level of the penoscrotal junction. There was no skin tethering, ulceration, or redness. He underwent routine investigations; complete blood count, liver function tests, renal function tests, human chorionic gonadotropin, and alpha fetoprotein were within the normal range. Magnetic resonance imaging has revealed a mass in the left inferolateral side of the base of the penis, measuring 3.1 cm in maximum dimension. The corpus cavernosa and corpus spongiosum are both unremarkable (Figure 1). The patient underwent surgical resection of the mass. The histopathologic examination revealed a neoplastic tissue composed of monophasic spindle cells arranged in intersecting fascicles and devoid of necrosis (Figure 2).

**Figure 3:**
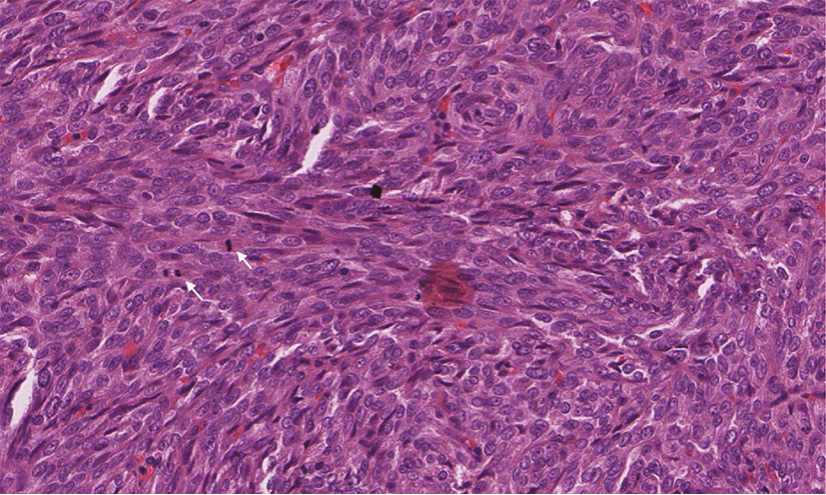
The lesion is mitotically active; the mitotic count is 3/10 HPF.

The lesion is mitotically active; the mitotic count is 3/10 HPF (Figure 3).

**Figure 4:**
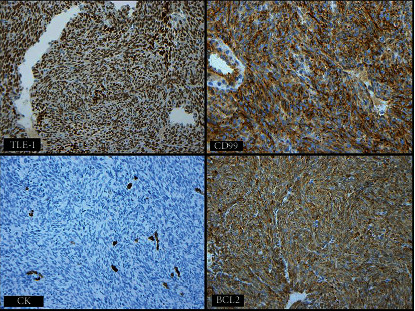
The lesion stained positive for pancytokeratin, AE1/AE3, TLE-1, CD99, and BCL-2.

The lesion stained positive for pancytokeratin, AE1/AE3, epithelial membrane antigen (EMA), TLE-1, CD99, and BCL-2, whereas negative for smooth muscle actin, desmin, S100, calretinin, CK5/6, and CD34 (Figure 4).

**Figure 5:**
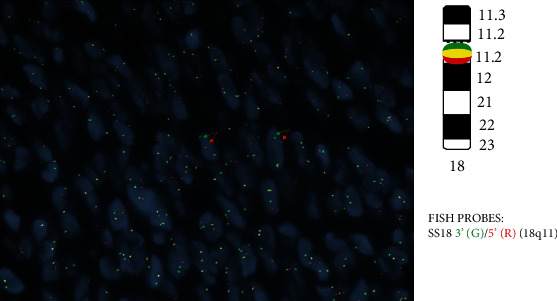
Break-apart FISH for SS18. Arrows indicate the separation of 3′SS18 and 5′SS18 probes.

Based on radiological, histopathological, and immunohistochemical findings, the patient was diagnosed with primary synovial sarcoma. The specimen was sent for molecular testing to the Mayo Clinic, which revealed a rearrangement involving the SS18 gene at 18q11.2 (Figure 5).

A month later, the patient underwent another surgery to evaluate the margins, and the microscopic examination showed no residual tumor. 17 months later, a computed tomography scan showed metastasis to the liver, first lumbar vertebrae, and right lower lobe of the lung. The patient received systemic chemotherapy with both ifosfamide and doxorubicin. Initially, he responded with a decrease in lung nodule size and an improvement in the hepatic lesion. However, a follow-up scan revealed an increased size of the hepatic lesion, so surgery was decided to resect the lesion, followed by radiation for the spine and lung lesions. The partial hepatectomy specimen revealed a metastatic sarcoma consistent with synovial sarcoma.

## 3. Discussion

Soft tissue sarcomas of the urogenital region are rare, only accounting for 2.1% of all soft tissue sarcomas and 1% to 2% of all malignant genitourinary tumors [9, 10]. The most common histological subtypes are leiomyosarcoma, liposarcoma, and, rhabdomyosarcoma [11–13]. A particular focus of this case report is synovial sarcoma of the urogenital system, which is extremely rare and has an unknown incidence in the literature. These tumors are often misdiagnosed as chronic inflammatory masses, squamous cell carcinomas, or metastatic tumors [14]. Primary urogenital synovial sarcoma mainly affects the kidney [15–18], prostate [19–21], and testis [22]. Very few case reports were retrieved that discussed the primary synovial sarcoma of the penis [14] and spermatic cord [23]. The literature review of the current report showed only one reported case of primary synovial sarcoma in the paratesticular area. To the best of our knowledge, this case report represents the second reported case in the literature, as Madhu and Venugopal reported the first case of a 28-year-old man who complained of a swelling in the right inguinoscrotal region [24]. Similar to our case, the examination revealed a smooth swelling without ulcers, tethering, or redness with no constitutional or urinary symptoms. Beyond the clinical aspects, the article did not provide additional information on pathological findings, immunohistochemical profile, or whether a molecular test was performed to confirm the presence of translocation t (X; 18) (SYT; SSX).

The current case underwent a microscopic examination and revealed monophasic spindle cells arranged in intersecting fascicles and devoid of necrosis (Figure 2). The lesion is mitotically active, with a mitotic count of 3/10 HPF (Figure 3). Immunohistochemically, the lesion stained positive for pancytokeratin, AE1/AE3, epithelial membrane antigen (EMA), TLE-1, CD99, and BCL-2, whereas it was negative for smooth muscle actin, desmin, S100, calretinin, CK5/6, and CD34. Molecular testing performed at the Mayo Clinic has revealed a rearrangement involving the SS18 gene at 18q11.2. These findings are consistent with the diagnosis of primary synovial sarcoma. These histopathologic and immunohistochemical findings are similar in most cases reported in the urogenital region and reiterate our case findings. However, only a few case reports performed a cytogenetic analysis for a definitive diagnosis.

The differential diagnosis of synovial sarcoma includes sarcomatoid carcinoma, malignant peripheral nerve sheath tumor, mesothelioma, malignant melanoma, and metastatic tumors. If histology and immunohistochemistry seem insufficient to establish the diagnosis, molecular testing is strongly recommended.

In light of the limited data reported on the treatment of urogenital sarcomas in general and synovial sarcomas in particular, it would seem appropriate to treat these rare neoplasms by adequate local excision with negative margins [5]. Due to the possibility of microscopic tumor seeding, surgical biopsy sites must be included in the resection field [25]. Due to the rarity of these tumors and the lack of reliable data, radiation therapy and adjuvant chemotherapy have been a source of controversy.

The natural history of paratesticular synovial sarcoma is unknown, and further cases need to be reported to establish an effective therapeutic regimen.

## 4. Conclusion

Synovial sarcoma is a malignant spindle cell soft tissue tumor of uncertain histogenesis. They represent 5–10% of all soft tissue sarcomas and commonly occur in the extremities, and they can also occur at other unusual sites. Primary scrotal paratesticular synovial sarcomas are rare tumors. Surgical excision remains the mainstay of treatment. These neoplasms require longer follow-up due to the risk of late recurrence and the development of distant metastases. Surgical resection of distant metastases is feasible.

## Data Availability

The authors can confirm that all relevant data are included in the article and its supplementary information files.
